# Essay upon the Methodical Treatment of Certain Diseases of the Womb by Vaginal and Uterine Injections

**Published:** 1841-07

**Authors:** 


### ART. XVIII.

*Essai sur un Traitement méthodique de quelques Maladies de la Matrice. Injections intra-vaginales et intra-utérines.* Par AUG. VIDAL (DE CASSIS), &c. &c.—*Paris*, 1840. 8vo, pp. 60.

*Essay upon the methodical Treatment of certain Diseases of the Womb by Vaginal and Uterine Injections.* By AUG. VIDAL, &c. &c.—*Paris*, 1840.

FOR the last ten years M. Vidal has strenuously opposed what he conceives to be the abuses committed in amputating the cervix uteri. He has taken great pains to investigate diseases of the uterus, both in his hospital and private practice; and in this brief essay he gives us the views he has formed, and a description of the practice he has found successful. The chief point to which he directs our attention is the employment of intra-uterine injections, which he considers very valuable in certain diseases of the uterus. He is aware that many are of a different opinion; that these injections have often been unsuccessfully employed; and that very serious objections have been urged against their use. He has deliberately reflected upon the arguments against him, and he concludes that the different results of the treatment he advocates in the hands of others, and the different opinions they have formed arise from the following causes: 1st, Some difference in the mode of using the remedy; 2d, Errors in diagnosis; 3d, A mistaken interpretation of the phenomena produced by the injections. M. Vidal first gives a coup d'œil of those uterine diseases which especially require injections, and he carefully describes the exact practice he has adopted in the hospital for women at Lourcine, that others may apply it in the proper way.

Leucorrhœa forms the first subject of the essay. The various local lesions that produce the discharge in this disease are pointed out, as well as the constitutional maladies which aggravate and render the discharge so tedious and difficult of removal, and which require general remedies, as well as local applications to relieve the disease. The anatomical characters of the diseases to which M. Vidal applies his treatment are either, 1st, engorgement; 2d, redness; 3d, ulcerations. The first are either acute or chronic, and the uterus itself is generally affected. Where redness exists the cervix uteri is always more or less enlarged. It either precedes or follows ulceration. If it follows ulceration, irregular spots are found upon the cervix, resembling those which are seen after chancres or pustules. The degree of redness varies under different circumstances. The ulcerations are either simple or granular. The former are very superficial, and exist with or are the consequence of inflammation of the vagina, which often extends to the uterus, and even to the ovaria. Granular ulceration is deeper, and presents the appearance of a suppurating wound. This form of ulceration is much more obstinate than the superficial, and is more common in women who have borne children. The os uteri is generally first affected with it, and "frequently in raising one lip of the os with the forceps an ulceration is detected which was not expected." The discharge arising from the ulceration varies in consistence and colour. Therefore the term *whites,* "fleur blanche," is not always applicable, for the discharge is often yellowish or greenish, and sometimes purulent and opaque, or at others, mucous and transparent. The more transparent the discharge is, the less serious is the disease. The following is the treatment adopted at Lourcine:

*Vaginal Injections.* The injection consists of a very concentrated decoction of walnut leaves, of the temperature of the room whatever may be the season. The instrument employed is a large clyster syringe. The speculum with two valves is first applied for the purpose of bringing the cervix uteri into view, upon which the stream of injection is thrown with as much force as the assistant can urge the piston. Immediately afterwards a plug of lint is placed upon the cervix, which imbibes the liquid that has remained in the vagina. The liquid thus injected presses upon the cervix and lowers its temperature; it acts also as an astringent. It rarely happens that the injection gives any pain when it is employed; but when the patient is put to bed, pains like colic around the hypogastrium and iliac regions sometimes arise. M. Vidal has frequently observed these pains when the cure has commenced, and so far from fearing them he looks upon them as favorable. These vaginal injections have frequently hastened in a very marked manner the cure of long-standing engorgements, as well as the healing of very obstinate ulcerations. The injections are repeated twice a week. They are omitted during the menstrual period. They are not employed during pregnancy, nor for three months after labour or miscarriage. If the cervix is very red and turgescent, leeches are applied to the anus, and hip baths or entire baths complete the treatment.

*Uterine Injections.* Some discharges resist the vaginal injection, as sometimes there are lesions of the internal surface of the cervix and of the uterus which are not reached by it. In these cases M. Vidal injects the uterus itself; and for the purpose of proving that this practice may be adopted without a risk, a number of experiments were tried, of which a detailed account is given. The mode of throwing injections into the uterus is also described, and especial cautions are given that the rules laid down may be strictly followed. In a former Number* we have noticed the dangers that occasionally arise from injecting the cavity of the uterus, and we therefore deem it proper to state briefly the effects observed by M. Vidal in his extensive practice. He found that in the majority of cases no pain was caused when the injection was used. In two instances only severe pain was caused, and in these the injections were discontinued. In some cases pains like colic occurred about an hour after the injection was used, which quickened the pulse and produced symptoms of reaction which alarmed the practitioner. But the occurrence of these pains was a rare exception, and did not happen once in ten times. In a brief appendix M. Vidal replies to certain objections which have been urged against the treatment he recommends.

* Br. and For. Med. Rev., vol. XI. p. 179.

